# Somatic nuclear blebbing in *Caenorhabditis elegans* is not a feature of organismal aging but a potential indicator of germline proliferation in early adulthood

**DOI:** 10.1093/g3journal/jkad029

**Published:** 2023-02-03

**Authors:** Qiang Fan, Xue-Mei Li, Chao Zhai, Bin Li, Shang-Tong Li, Meng-Qiu Dong

**Affiliations:** Chinese Academy of Medical Sciences & Peking Union Medical College, Beijing 100730, China; National Institute of Biological Sciences (NIBS), Beijing 102206, China; Tsinghua Institute of Multidisciplinary Biomedical Research, Tsinghua University, Beijing 102206, China; National Institute of Biological Sciences (NIBS), Beijing 102206, China; Tsinghua Institute of Multidisciplinary Biomedical Research, Tsinghua University, Beijing 102206, China; National Institute of Biological Sciences (NIBS), Beijing 102206, China; Tsinghua Institute of Multidisciplinary Biomedical Research, Tsinghua University, Beijing 102206, China; National Institute of Biological Sciences (NIBS), Beijing 102206, China; Tsinghua Institute of Multidisciplinary Biomedical Research, Tsinghua University, Beijing 102206, China; National Institute of Biological Sciences (NIBS), Beijing 102206, China; Tsinghua Institute of Multidisciplinary Biomedical Research, Tsinghua University, Beijing 102206, China; Chinese Academy of Medical Sciences & Peking Union Medical College, Beijing 100730, China; National Institute of Biological Sciences (NIBS), Beijing 102206, China; Tsinghua Institute of Multidisciplinary Biomedical Research, Tsinghua University, Beijing 102206, China

**Keywords:** aging, *C. elegans*, nuclear morphology, chromatin loss, proliferative germ cells

## Abstract

Abnormal nuclear morphology is suggested to be a hallmark of aging and one such abnormality is nuclear blebbing. However, little is known about whether and how nuclear blebbing participates in animal aging, and what regulates it. In this study, we show that the frequency of nuclear blebbing in the hypodermis increases during aging in wild-type *C. elegans*. These nuclear blebs are enveloped by the nuclear lamina, the inner and the outer nuclear membrane, and 42% of them contain chromatin. Although nuclear blebbing could lead to DNA loss if chromatin-containing blebs detach and fuse with lysosomes, we find by time-lapse imaging that nuclear blebs rarely detach, and the estimated lifetime of a nuclear bleb is 772 h or 32 days. The amount of DNA lost through nuclear blebbing is estimated to be about 0.1% of the total DNA loss by adult Day 11. Furthermore, the frequency of nuclear blebbing does not correlate with the rate of aging in *C. elegans*. Old age does not necessarily induce nuclear blebbing, neither does starvation, heat stress, or oxidative stress. Intriguingly, we find that proliferation of germ cells promotes nuclear blebbing.

## Introduction

In eukaryotic cells, the nucleus is enclosed by the nuclear envelope, which consists of two lipid bilayer membranes, the outer nuclear membranes (ONM) and the inner nuclear membranes (INM). The two membranes are fastened up by the Linker of Nucleoskeleton and Cytoskeleton (LINC) complexes. LINC complexes are composed of KASH domain containing-proteins anchored to ONM and SUN domain containing-proteins anchored to INM (Fridkin *et al.* 2009). INM is lined with the nuclear lamina, a meshwork of mostly lamins (Liu *et al.* 2000), which bind to each other and also to INM proteins such as emerin (EMR-1 in *C. elegans*) (Fairley *et al.* 1999; Clements *et al.* 2000; Lee *et al.* 2000) and LEM domain protein 2 (Brachner *et al.* 2005). Scattered over the nuclear envelope are Nuclear Pore Complexes (NPCs), which are composed of nucleoporins, and regulate material exchange between the nucleoplasm and the cytoplasm (Cohen-Fix and Askjaer 2017).

Normally, nuclei are oval-shaped, but abnormalities in nuclear morphology arise during aging (Zink *et al.* 2004; Scaffidi and Misteli 2006; Schreiber and Kennedy 2013). Abnormal nuclear morphology is suggested to be a hallmark of aging (D'Angelo *et al.* 2009; Robijns *et al.* 2018; Martins *et al.* 2020; Pathak *et al.* 2021) and cellular senescence (Heckenbach *et al.* 2022). Convolution of the nuclear membrane or nuclear lamina, detected in most cases using a fluorescently labeled lamin protein, is a visually striking, gross abnormality of the nuclear shape and is referred to here as nuclear membrane convolution for simplicity. Nuclear membrane convolution is a characteristic of fibroblasts isolated from normally aged humans (Scaffidi and Misteli 2006). In aged *C. elegans,* the nuclear membrane also becomes convoluted (Haithcock *et al.* 2005). Furthermore, age-associated nuclear membrane convolution is slowed down in the *daf-2(e1370)* mutant (Haithcock *et al.* 2005; Zhao *et al.* 2017) and the *eat-2(ad1116)* mutant (Charar *et al.* 2021), both are long-lived compared to the wild-type worm. However, pharmacological inhibition of farnesylation of lamin proteins, which ameliorates age-associated nuclear membrane convolution, fails to extend *C. elegans* lifespan (Bar *et al.* 2009; Bar and Gruenbaum 2010). This suggests that nuclear membrane convolution can be uncoupled from aging. However, other types of nuclear abnormalities such as nuclear blebbing have not been examined from this perspective.

Relative to nuclear membrane convolution, which is a global deformation of the nucleus, nuclear blebbing is a localized deformation, which involves the formation of relatively small protrusions from the surface of a nucleus (Stephens *et al.* 2018). Nuclear blebs can be seen on otherwise smooth, oval-shaped nuclei of young adult animals. It has been reported that nuclear blebs in cultured senescent cells can mediate the nucleus-to-cytoplasm transport of chromatin and lamin B1 (Ivanov *et al.* 2013; Dou *et al.* 2015; Dou *et al.* 2017). The abnormal presence of chromatin in the cytoplasm activates the cGAS-STING (cyclic GMP–AMP synthase linked to stimulator of interferon genes) pathway, which senses cytosolic DNA and in turn, promotes secretion of pro-inflammatory cytokines, a key feature of senescent cells (Dou *et al.* 2017). Additional studies show that ectopic lamin B1 in the cytoplasm is targeted to the lysosome for degradation, and the loss of lamin B1 promotes cellular senescence (Ivanov *et al.* 2013; Dou *et al.* 2015). Thus, nuclear blebbing may mediate cellular senescence.

Nuclear blebs have been observed during aging in *C. elegans*, but remain poorly characterized (Haithcock *et al.* 2005). Using *C. elegans* as an aging model, we try to find out in this study: 1) whether the nuclear membrane surrounding a nuclear bleb is of an intact structure with normal ONM, INM, nuclear lamina and nuclear pores; 2) whether chromatin is present inside a nuclear bleb and whether blebbing leads to degradation of chromatin in the cytoplasm as has been reported in cultured cells; 3) how nuclear blebbing changes with age; and 4) whether nuclear blebbing is coupled with organismal aging. Below we report our findings for these questions, and an additional intriguing discovery that nuclear blebbing in the hypodermis responds to proliferating germ cells in the gonad.

## Materials and methods

### C. elegans strains

The following strains were used in this work:

UD484 (*yc32*[*gfp::lmn-1*] I) (Bone *et al.* 2016)

MQD2907 (*yc32*[*gfp::lmn-1*] I; *bqSi226*[P*emr-1::emr-1::mCherry + unc-119(+)*] IV)

MQD2908 (*bqSi226*[P*emr-1::emr-1::mCherry + unc-119(+)*] IV; *hqIs466*[*npp-6::gfp*])

MQD2615 (*yc32*[*gfp::lmn-1*] I; *thu7*[*his-72::mcherry*])

MQD1844 (*p720–4*[P*lmn-1::emr-1::gfp::unc-54 3'UTR + unc-119(+)*]; *thu7*[*his-72::mcherry*])

MQD2029 (*thu7*[*his-72::mcherry*]; *qxIs430*[P*scav-3::scav-3::gfp*])

MQD1807 (*thu7*[*his-72::mcherry*]; *qxIs520*[P*vha-6::laat-1::gfp*])

MQD2974 (*bqSi226*[P*emr-1::emr-1::mCherry + unc-119(+)*] IV; *qxIs430*[P*scav-3::scav-3::gfp*])

MQD2808 (*yc32*[*gfp::lmn-1*] I; *daf-2(e1370)* III)

MQD2917 (*glp-1(e2141)* III; *yc32*[*gfp::lmn-1*] I)

MQD2918 (*fer-15(b26ts)* II; *yc32*[*gfp::lmn-1*] I)

LW699 (*p720–4*[P*lmn-1::emr-1::gfp::unc-54 3'UTR + unc-119(+)*]) [11]

MQD2423 (*bqSi235* [P*emr-1::emr-1::GFP + unc-119(+)*] II)

MQD2425 (*bqSi226* [P*emr-1::emr-1::mCherry + unc-119(+)*] IV)

MQD2684 (*hqKi450*[*emr-1::gfp*] I)

### Plasmid

Plasmids used to construct GFP::GFP::KASH knock-in strain, GFP::GFP::KASH was inserted into the single copy insertion site on chr II:

GGKKI-HR (carrying repair template for homology directed repair, modified from pPD95_77)

GGKKI-sg1 and GGKKI-sg2 (carrying sgRNA, modified from pDD162)

### *C. elegans* maintenance condition

Worms were maintained at 20°C on Nematode Growth Medium (NGM) plates seeded with live *E. coli*(OP50) unless otherwise indicated.

*glp-1(e2141)* mutants *fer-15(b26)* mutants were maintained at 15°C. To induce temperature-sensitive sterile phenotype, these two mutants were cultured at 25°C since embryonic stage.

### Confocal imaging

All images were acquired with a spinning-disk confocal microscope (UltraVIEW VOX; PerkinElmer) equipped with a 63×, 1.4 numerical aperture oil-immersion objective. Worms were placed on 3% agarose pads and anesthetized with 10 mM levamisole solution. The exposure time and laser power were varied to balance the fluorescence intensity among samples.

For time-lapse imaging, room temperature was kept at 20°C and worms were imaged every 30 mins for 10 hours.

Nuclear blebs were identified manually.

### Scanning electronic microscopy analysis

EM samples and Scanning EM imaging were conducted according to the methods developed by Li et al (Li *et al.* 2017). Worms at adult Day 2, Day 9, and Day 18 were collected and were fixed via high-pressure freezing (Wohlwend HPF Compact-01). Fixed samples were put into 1% OSO4 acetone solution for dehydration (Leica AFS2), and the temperatures control program was set as −90°C for 72 hrs.; increase 2°C per hour until reach −60°C; −60°C for 10 hrs.; increase 2°C per hour until reach −30°C; −30°C for 10 hrs.; increase 2°C per hour until reach 4°C. Staining samples with UAc saturated acetone solution for 3.5 hrs. at room temperature. Use SPI-PON812 resin (SPI-CHEM) for filtration, then samples were embedded and polymerized at 60°C for 48 hrs. 70 nm sections sectioned by ultramicrotome (UC6, Leica, Germany) were collected on PE tap (Shanghai Jinghou Electronics Technology Co., Ltd. China) and stuck on a silicon wafer (Suzhou crystal silicon electronic & technology Co., Ltd. China) via electroconductive adhesive tape (NISSHIN EM Co. Ltd, Japan). Sections were examined via SEM (FEI Helios NanoLab 600i) equipped with CBS detector with 2.0 kV in accelerating voltage, 0.69 nA in current, 10 μs in dwell time, and visualized via the software, xT microscope control (FEI, version 5.2.2.2898) and PinPoint (developed by Li et al. (Li *et al.* 2017)).

### Real-time PCR

At each time point, 50 worms were collected into 50 μl of lysis buffer and frozen immediately in liquid nitrogen. Lysis was carried out by incubation at 60°C for 1 h and proteinase K was inactivated by incubating at 95°C for 10 min. RT-PCR was carried out on an ABI 7,500 Fast Real-Time PCR System using a TAKARA real-time PCR kit (SYBR Premix Ex TaqTM II). The primers used were as follows:

*ced-7* chrIII F: TGCACATGTCGTTATGGCTT,

R: AAGCAGCAGGACTCACGAAT

*cct-1*chrII F: CAAGGGACCGAAATCTCGTA,

R: CAGAGTGAGTCGTGAACCGA

*fat-3* chrIV F: ATCCAATACAGGTCGATGGC,

R:CAGCTCCTCCTGGATGTTTC

*pmp-3* chrV F: TTGTCACCCCGGGAAGCAGA,

R: GGCAACAAAACGACGGAAGGAA

*rDNA(26S)* chrI F: TTGAACGGCCCTTAAAACACCA,

R: TTGCCGACTTCCCTTACCTACATT

### Quantitative western blot analysis

For measuring H3 protein levels, *glp-1(e2141)* mutants were hatched and cultured at 25°C. At each timepoint, 100 worms were collected into 20 μl M9 buffer, flash-frozen and stored at −80°C. The worms were thawed, mixed with 20 μl 2 × SDS loading buffer, boiled for 15 min at 100°C before loading. Blots were incubated with anti-H3(1:5000, Cat#1791, Abcam) or anti-tubulin (1:5,000 dilution, Cat#T3526, Sigma-Aldrich). Quantification was carried out using ImageJ.

### Stress condition

For heat stress, on AD2, worms were exposed to 37°C for 2 h and imaged immediately; for starvation stress, on AD2, worms were transferred to NGM plates containing no OP50 and imaged 10 hours after starvation initiation.

For paraquat (PQ) stress, worms were hatched and cultured on NGM plates containing live OP50 and 0.1 mM or 2 mM PQ and imaged on AD3.

### γirradiation

For irradiation, on AD2, worms were exposed to 80 Gy irradiation using a Gammacell 1,000 blood irradiator and imaged 10 hours later.

### 5'-fluorodeoxyuridine (FUDR) treatment

For FUDR treatment, 6-cm Nematode Growth Medium (NGM) plates containing 100 μM FUDR were seeded with 200 μl concentrated OP50. Worms were transferred to FUDR plates since mid L4 stage.

### Thioflavin t (ThT) treatment

For ThT treatment, 6-cm NGM plates containing 100 μM ThT were seeded with 200 μl concentrated OP50. Worms were hatched and cultured on ThT plates.

## Results

### Nuclear blebbing frequency increases during aging in wild-type *C. elegans*

**Fig. 1.**
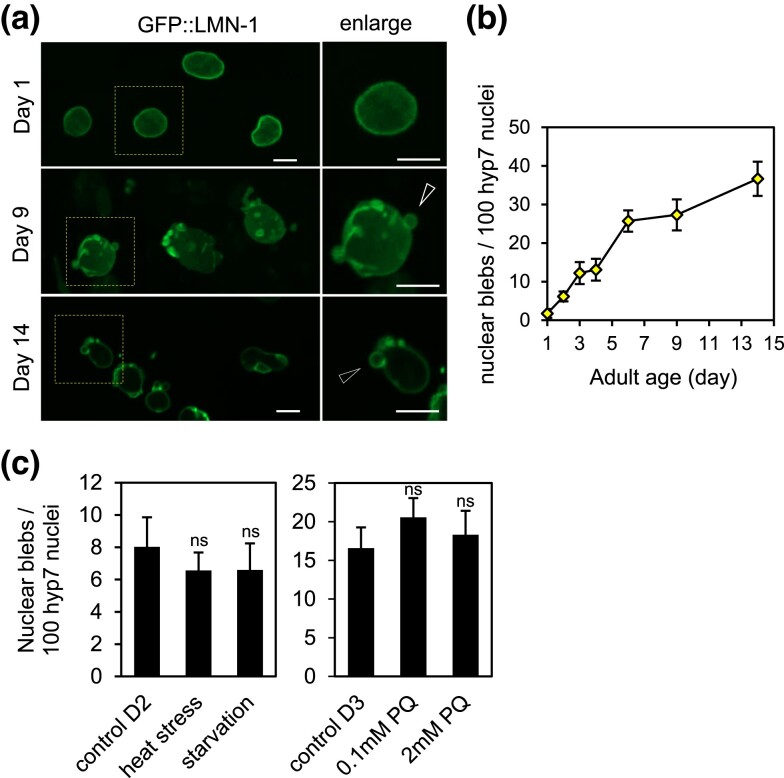
Nuclear blebs accumulate during aging in *C. elegans.* a) Nuclei of hyp 7 on adult Day 1, 9, and 14. The boxed regions are magnified and shown on the right, with arrowheads pointing to nuclear blebs. b) Age-dependent increase of nuclear blebs. c) The indicated stress conditions did not induce nuclear blebbing. Adult Day 2 (D2) worms were subjected to heat stress and starvation, and adult Day 3 (D3) worms were treated with paraquat (PQ). ns, not significant (*P* > 0.001), as determined by Student's T-test. Error bars represent standard error. Scale bars represent 5 µm. The hyp 7 nuclei of the *gfp::lmn-1* knock-in strain UD484 were imaged and analyzed in (a-c).

We started out by characterizing nuclear blebs in aged *C. elegans.* We chose to focus on hypodermal nuclei, specifically, the nuclei of the hyp7 cell, which wraps around most of the worm body. Large and thin, hyp7 has a total of 139 nuclei, a product of extensive cell-cell fusion during embryonic and larval development (Shemer and Podbilewicz 2000). The hyp7 nuclei are relatively large, uniform in appearance, and close to the body surface, thus yield high image quality. LMN-1 is the sole lamin protein in *C. elegans*. By imaging a knock-in worm strain expressing GFP::LMN-1 from the endogenous *lmn-1* locus, we observed that hyp7 nuclei from young animals had few blebs whereas those from aged worms had many (Fig. 1a). The frequency of nuclear blebbing increased with age, from close to zero on adult Day 1 (AD1) to about 35 blebs per 100 hyp7 nuclei on AD14 (Fig. 1b). Neither heat stress, starvation, nor paraquat treatment, which produces reactive oxygen species (ROS) in the cell (Shen *et al.* 2014), increased the frequency of nuclear blebbing (Fig. 1c), suggesting that nuclear blebbing is a rather specific phenomenon associated with old age, not something induced by stress in general.

### Characteristics of nuclear blebs

**Fig. 2.**
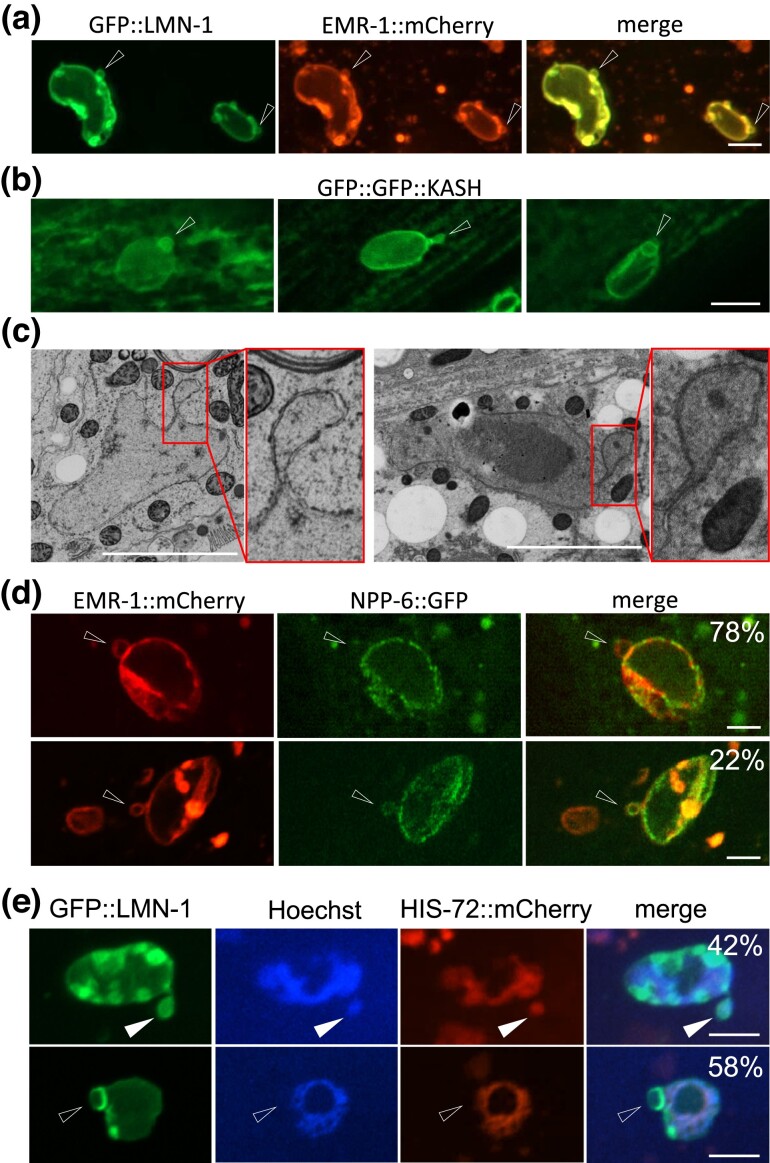
Characteristics of nuclear blebs in aged *C. elegans.* a) Double labeling of nuclear blebs by a nuclear lamina marker GFP::LMN-1 and an INM marker EMR-1::mCherry. b) Labeling of nuclear blebs by an ONM marker containing a tandem GFP tag fused with the KASH domain of UNC-83. c) Two EM images of nuclear blebs. Selected regions (smaller boxes) are magnified and shown as insets (larger boxes) (D) 78% nuclear blebs marked by EMR-1::mCherry were not labeled by NPP-6::GFP (upper row), but 22% were (lower row). e) On adult Day 4, 42% of the nuclear blebs contained chromatin (upper, filled arrowhead) and 58% not (lower, empty arrowhead). Scale bars, 5 µm.

Before examining the relationship between nuclear blebbing and *C. elegans* aging, we asked whether nuclear blebs are enveloped by normal nuclear membrane. We used EMR-1::mCherry to mark INM and examined it with respect to GFP::LMN-1, and found that they colocalized in all hyp7 nuclear blebs examined (Fig. 2a). Nuclear blebs were also positive for GFP::GFP::KASH, a marker of ONM with a tandem GFP tag (Fig. 2b). Electron microscopy (EM) analysis confirmed the presence of ONM and INM in nuclear blebs (Fig. 2c). We then asked whether nuclear blebs also possess nuclear pores as the mother nuclei do. NPP-6, the *C. elegans* ortholog of human nucleoporin 160, is a peripheral subunit of NPC of multiple copies (16 copies per NPC) (Tie *et al.* 2016). We tagged NPP-6 with GFP to label nuclear pores and found that 78% of the nuclear blebs (*n* = 37) marked by EMR-1::mCherry contained no NPP-6::GFP, suggesting that nuclear blebs are deficient in NPCs or nuclear pores (Fig. 2d).

We further asked whether nuclear blebs contain chromatin. Using HIS-72::mCherry to indicate the presence of histones and the Hoechst dye to mark DNA, we found that 42% of the nuclear blebs contained both HIS-72::mCherry and DNA, whereas 58% had neither (*n* = 45) (Fig. 2e). This intriguing result indicates that less than 50% of nuclear blebs contain chromatin.

In summary, we find that *C. elegans* hyp7 nuclei gradually form and accumulate nuclear blebs as the animal grows old. These blebs have ONM, INM and LMN-1, the marker of nuclear lamina. 42% of them contain chromatin.

### Nuclear blebbing is a minor contributor to chromatin loss in aged *C. elegans*

It has been shown that the DNA copy numbers per worm decreased with the age of the animal (Golden *et al.* 2007). Here we repeated the experiment and verified the finding on each of the five representative genes (one encoding rRNA and four coding proteins, each on a different chromosome) (Supplementary Fig. 1a). The protein level of histone H3 also decreased gradually during aging (Supplementary Fig. 1b).

**Fig. 3.**
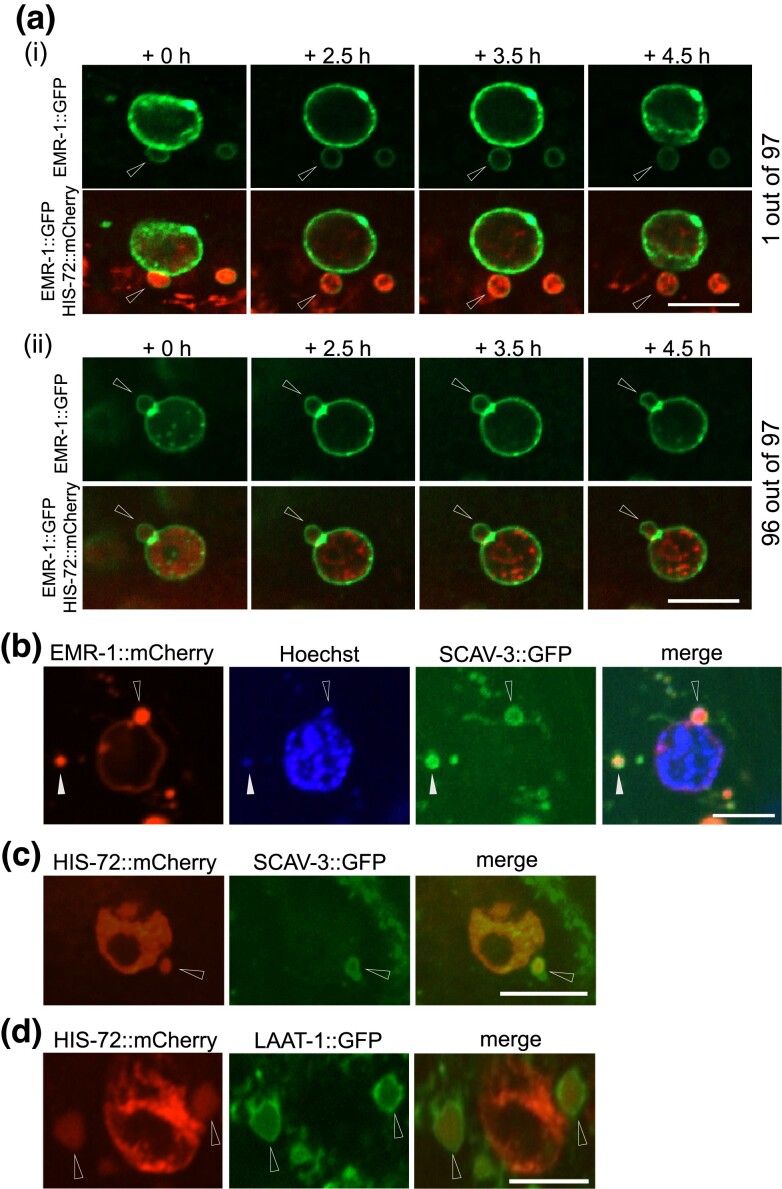
Very rarely, nuclear blebs detach and give rise to cytoplasmic chromatin. a) (i) detachment of a nuclear bleb dually labeled by EMR-1::GFP and HIS-72::mCherry, representing 1 of 97; (ii) a nuclear bleb that did not detach from the parent nucleus, representing 96 of 97; arrowheads indicate nuclear blebs. b) Cytoplasmic chromatin captured by lysosome; EMR-1::mCherry, marker of INM; Hoechst, marker of DNA; SCAV-3::GFP, marker of lysosome; arrowheads indicate cytoplasmic chromatin. (C, D) A subpopulation of cytoplasmic chromatin was found in lysosomes marked by either SCAV-3::GFP (C) or LAAT-1::GFP (D); arrowheads indicate cytoplasmic chromatin. Scale bars, 5 µm.

Next, we asked whether nuclear blebbing contributes to chromatin loss. Using time-lapse imaging, we tracked 97 nuclear blebs each for 8 h and found only one that detached from the parent nucleus during the observation period (Fig. 3a and Supplementary File 1). Thus, although nuclear blebbing can give rise to cytoplasmic chromatin, such events are rare in *C. elegans*. The estimated lifetime of a nuclear bleb is ∼772 hours and the corresponding half-life of nuclear blebs is ∼535 hours (Supplementary File 2).

To find out whether lysosomes degrade cytoplasmic chromatin in *C. elegans* as has been reported in cultured senescent cells (Ivanov *et al.* 2013), we examined the colocalization of cytoplasmic chromatin with two fluorescently labeled lysosomal membrane proteins SCAV-3 and LAAT-1. Using the nuclear membrane marker EMR-1::mCherry, we observed some EMR-1::mCherry puncta that were either attached to (Fig. 3b, indicated by empty arrow head) or separated by a short distance from the nucleus (Fig. 3b, indicated by filled arrow head). These puncta contained Hoechst-stained DNA, and were wrapped by SCAV-3::GFP, indicating that they were derived from nuclear blebs and then captured by lysosomes. We also observed cytoplasmic structures positive for HIS-72::mCherry and SCAV-3::GFP (Fig. 3c) or HIS-72::mCherry and LAAT-1::GFP (Fig. 3d), some of which positioned right next to the nucleus. The size and the position of such histone containing lysosomes invite comparison with detached nuclear blebs. Together, these observations indicate that chromatin-containing nuclear blebs could be engulfed by lysosomes.

Next, we considered a plausible hypothesis that nuclear blebbing may mediate degradation of damaged chromatin. Previous studies have shown that nuclear blebs in cultured cells are enriched with γ-H2AX, a classic marker of DNA double-strand breaks (Ivanov *et al.* 2013; Karoutas *et al.* 2019). As the *C. elegans* genome does not encode γ-H2AX, we did not examine directly whether damaged chromatin was enriched in nuclear blebs. Instead, we induced DNA damage by subjecting *C. elegans* to ionizing irradiation and expected the frequency of nuclear blebbing to spike after irradiation if nuclear blebbing were a significant mechanism for the animal to remove damaged chromatin. However, this was not the case (Supplementary Fig. 1c).

Using mathematical modeling, we estimated the contribution of nuclear blebbing to chromatin loss. By adult Day 14, the estimated number of detached blebs for each nucleus is predicted to be 0.049 (Supplementary File 2). About 42% nuclear blebs contained DNA (Fig. 2e), and the diameter and the deduced volume of a nuclear bleb are roughly 1/5 and 1/125 of that of the parent nucleus (Fig. 1a). Assuming that a DNA-containing nuclear bleb contains about 1/125 or 0.8% of the nuclear DNA, then it can be estimated that by adult Day 11, DNA loss through nuclear blebbing in hyp7 accounts for ∼ 0.016% of total nuclear DNA (0.049*42%*0.8%= 0.016%). By adult Day 11, the *C. elegans* soma loses 10–25% of total nuclear DNA (Fig S1A). If hyp7 also lose 10–25% of nuclear DNA, then about 0.1% (0.016%/15%= 0.1%) of that is lost through nuclear blebbing. We thus propose that nuclear blebbing is only a minor contributor to chromatin loss during *C. elegans* aging.

In summary, the above results demonstrate that in aged *C. elegans*, nuclear blebbing sporadically leads to separation of chromatin from the nucleus and thus formation of cytoplasmic chromatin, which is then targeted to lysosomes for degradation. Our data do not support the idea that nuclear blebbing is a major mechanism for the nucleus-to-cytoplasm transport of chromatin.

### The rate of nuclear blebbing does not correlate with that of aging in *C. elegans*

**Fig. 4.**
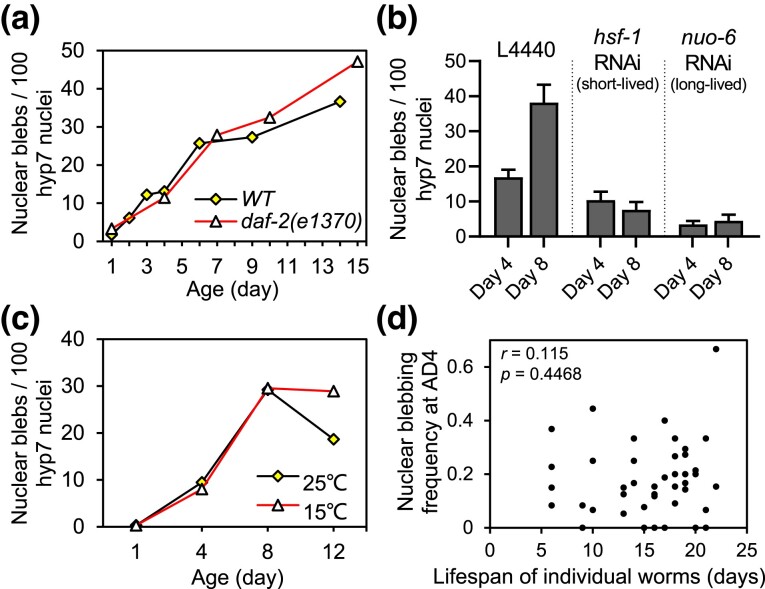
Uncoupling of the rate of aging and the rate of nuclear blebbing. a) The frequency of nuclear blebbing increased with age at essentially the same rate in the WT and the long-lived *daf-2(e1370)* mutant worms. Worms were maintained under the standard culture temperature of 20°C. b) *hsf-1* RNAi treatment, which shortens lifespan, did not accelerate nuclear blebbing. *nuo-6* RNAi treatment, which extends lifespan, inhibited nuclear blebbing. c)WT worms cultured at 15°C, which extends lifespan, and those cultured at 25°C, which shortens lifespan, displayed the same nuclear blebbing frequency from AD 1 to AD 8. d) No correlation between the lifespan of individual worms and the nuclear blebbing frequency measured on AD4. Spearman correlation, *r* = 0.115, *P* = 0.4468, two-tailed.

Next, we asked whether nuclear blebbing is modulated by, or even modulates, the rate of aging in *C. elegans*. The insulin/IGF-1-like signaling pathway regulates aging across animal species (Kenyon 2010). The *daf-2(e1370)* mutant *C. elegans,* in which the insulin/IGF-1 receptor gene is compromised, lives twice as long as the wild-type (Kenyon *et al.* 1993). However, in this long-lived *daf-2(e1370)* mutant, the frequency of nuclear blebbing is hardly different from that in wild-type worms for as long as two weeks into adulthood (Fig. 4a). In another longevity model generated by knocking down *nuo-6* (Yang and Hekimi 2010), which encodes a NADH ubiquinone oxidoreductase in the mitochondrial electron transport chain, the nuclear blebbing frequency is indeed lower than WT, but it is also lower in the short-lived *hsf-1* RNAi worms (Fig. 4b). The *hsf-1* gene encodes a highly conserved transcription factor Heat Shock Factor 1, which plays a critical role in maintaining proteostasis (Garigan *et al.* 2002). In sum, we find no correlation between nuclear blebbing and lifespan.

Temperature is a key environmental factor that controls lifespan of *C. elegans*. At elevated temperatures such as 25°C, worms age faster (Hosono *et al.* 1982). However, a five degree increase or decrease from the standard culture temperature of 20°C did not accelerate or decelerate nuclear blebbing (Fig. 4c).

In addition, there are significant inter-individual differences in the rate of aging. Even for a population of isogeneic worms cultured in the same environment, e.g. on the same plate, the lifespans of individual worms vary dramatically (Kinser *et al.* 2021). We found that among the wild-type worms kept under the same condition, the frequency of nuclear blebbing at adult Day 4 did not correlate with lifespan (Fig. 4d).

Thus, the results above suggest that in *C. elegans*, the rate of nuclear blebbing is not coupled with that of aging.

### Proliferating germline stem cells promote nuclear blebbing in hypodermal cells

**Fig. 5.**
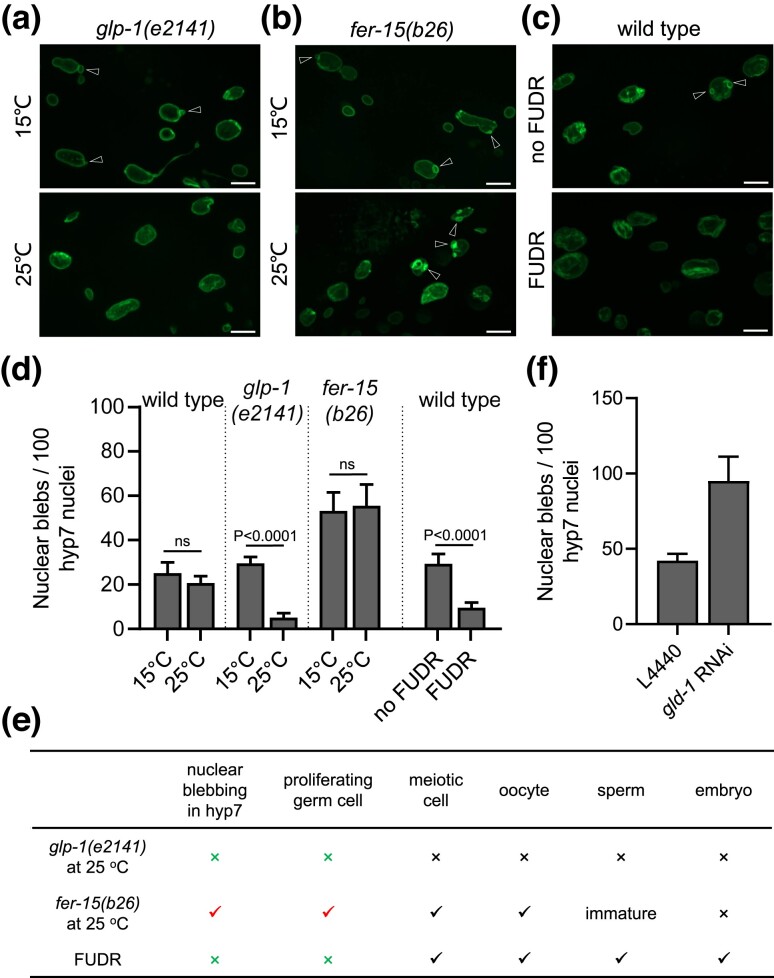
Proliferating germ cells promote nuclear blebbing in the hypodermis. (A, B, C) Representative images of nuclear blebs of *glp-1(e2141)*, *fer-15(b26)*, and WT worms on AD5. d) Nuclear blebbing frequency on AD5 under the indicated conditions. e) Summary of *glp-1(e2141)* at 25°C, *fer-15(b26)* at 25°C and the effects of FUDR on the germline and nuclear blebbing in the hypodermis. f) On AD5, knocking down *gld-1* promoted nuclear blebbing in the hypodermis. The *gfp::lmn-1* knock-in strain was used in (A-F). Scale bars, 10 µm. Arrowheads indicate nuclear blebs. Error bars represent standard error, and *P* values from Student's T-test are shown. ns, not significant (*P* > 0.00 l), as determined by Student's T-test.

Since nuclear blebbing is uncoupled from aging (Fig. 4) and is not induced by heat, starvation, and ROS (Fig. 1c), we wondered what causes nuclear blebbing. We found that nuclear blebbing in the hypodermis responds to the activity of the gonad. The temperature-sensitive *glp-1(e2141)* mutation causes germ cells to enter meiosis prematurely at the restrictive temperature of 25°C, resulting in an empty gonad of few germs cells and sterility; when cultured at 15°C, the *glp-1(e2141)* mutant has an intact gonad and reproduces normally (Austin and Kimble 1987; Priess *et al.* 1987). We noticed that for the *glp-1(e2141)* mutant, the frequency of nuclear blebbing in the hypodermis was markedly lower at 25°C than at 15°C (Fig. 5a), and subsequent quantification revealed a six-fold difference (∼5 and ∼30 blebs per 100 hyp7 nuclei on AD5, respectively, at 25°C and 15°C) (Fig. 5d). As a control, wild-type worms showed no significant difference in the frequency of nuclear blebbing between 25°C and 15°C (Fig. 5d). This result indicates that the mechanism that regulates nuclear blebbing of somatic cells is responsive to the activity of the reproductive system, not the culture temperature.

To further dissect the connection between somatic nuclear blebbing and the reproduction system, we analyzed the *b26ts* mutant of *fer-15*, also known as *rrf-3*. Although defective in spermatogenesis in a temperature-sensitive manner (Hirsh and Vanderslice 1976), the germ cells and oocytes of the *fer-15(b26ts)* worms are normal (Hirsh and Vanderslice 1976). Intriguingly, there was no significant difference in the frequency of nuclear blebbing between *fer-15(b26ts)* worms cultured at the restrictive temperature of 25°C and those at the permissive temperature of 15°C (Fig. 5b and 5d). Thus, nuclear blebbing in the hypodermis does not respond to the presence or absence of mature sperm, nor to embryos.

Next, we tested the effect of FUDR (5'-fluorodeoxyuridine), a DNA synthesis inhibitor that can prevent the proliferation of germ cells (Golden *et al.* 2007). When FUDR treatment was initiated at the mid L4 stage, we found that worms retained meiotic cells, oocytes, sperm and even embryos, but no proliferating germ cells (Fig S2 and 5E). Interestingly, the hyp7 nuclei of FUDR-treated worms accumulated fewer nuclear blebs than those of untreated worms (Fig. 5c, 5d and Supplementary 3).

Contrasting the above results, we find that the common denominator is evidently proliferating germ cells (Fig. 5d). Proliferation of germ cells is inhibited in FUDR-treated WT worms and in *glp-1(e2141)* mutant worms cultured at 25°C, and for both, hyp7 nuclear blebbing is also inhibited. Conversely, proliferation of germ cells is not affected in *fer-15(b26ts)* worms, neither is hyp7 nuclear blebbing (Fig. 5c-d). Further, the *gld-1* RNAi treatment, which caused extensive mitosis of germline stem cells, significantly promoted nuclear blebbing (Fig. 5f). Thus, we propose that germ cell proliferation of young, reproductive adult worms promotes nuclear blebbing in the hypodermis as observed on adult Day 5, when the egg-laying activity normally ends.

## Discussion

### Characteristics of nuclear blebbing in *c. elegans*

For more than half a century, the term “nuclear bleb” has been used to describe protrusions of the nuclear envelope into the cytoplasm under different biological conditions (Clark 1960; Ruddle 1962; Törő and Olah 1966; Ahearn *et al.* 1967; McDuffie 1967; Ahearn *et al.* 1974; Mishra and Munnet 1979; Szollosi and Szollosi 1988; Vigouroux *et al.* 2001; Dou *et al.* 2015; Furusawa *et al.* 2015). However, these “blebs” differ in morphology, composition, cause of formation, and function. This study shows that in aged *C. elegans*, nuclear blebs are enclosed by the nuclear lamina and the nuclear envelope, and less than 50% of them contain chromatin. This is in line with literature reports. Depending on the biological context, nuclear blebs may be deficient in LamB (Vigouroux *et al.* 2001; Furusawa *et al.* 2015), NPCs (Vigouroux *et al.* 2001; Karoutas *et al.* 2019), or RNA Pol II (Karoutas *et al.* 2019), or enriched with γ-H2AX (Ivanov *et al.* 2013; Karoutas *et al.* 2019) or heterochromatin (Karoutas *et al.* 2019).

### Nuclear blebbing plays a minor role in mediating chromatin loss during aging

*C. elegans* worms lose chromatin during aging (Supplementary Fig. 1 and (Golden *et al.* 2007)). Nuclear blebbing has been reported to mediate the nucleus-to-cytoplasm transport (Patterson *et al.* 2011; Speese *et al.* 2012; Li *et al.* 2016). About 42% of the nuclear blebs in aged *C. elegans* contain chromatin (Fig. 2e). However, only a tiny fraction of these blebs detach (Fig. 3a), and their contents can be degraded by lysosomes (Fig. 3b-d). Using mathematical modeling, we estimated the contribution of nuclear blebbing to chromatin loss (File S2). We reason that during aging, nuclear blebbing is a minor contributor to chromatin loss. Other biological processes, such as nuclei loss, may also play a part (Golden *et al.* 2007; McGee *et al.* 2011).

With respect to the nucleus-to-cytoplasm transport via nuclear blebbing, one may ask what cargos the chromatin-negative blebs (Fig. 2e) may contain. Our electron microscopy analysis did not reveal RNP-like granules (Li *et al.* 2016) in nuclear blebs of hypodermal cells.

### Proliferative germ cells regulate nuclear blebbing in the hypodermis

Surprisingly, inhibiting proliferation of germ cells prevented nuclear blebbing in hypodermal cells (Fig. 5). Exposure of worms to the amyloid-binding compound Thioflavin T (ThT) can promote proteostasis (Alavez *et al.* 2011). We observed that ThT-treated worms exhibited fewer blebs (Supplementary Fig. 4). These observations echo the previous findings that after worms reach sexual maturity, germline stem cells abruptly turn down the heat shock response of somatic tissues (Shemesh *et al.* 2013; Labbadia and Morimoto 2015). Considering the vital role of the heat shock response pathway in maintaining proteostasis, we propose that proliferative germ cells may promote somatic nuclear blebbing by disrupting proteostasis. However, knocking down *hsf-1* did not promote nuclear blebbing (Fig. 4b), arguing against this simple model. Further studies are needed to unveil the underlying mechanism.

### Nuclear blebbing and nuclear lamina convolution are regulated by distinct mechanisms

We propose that in aged *C. elegans*, nuclear blebbing and convolution of nuclear lamina are regulated by distinct mechanisms, based on the observations that: (1) convolution was slowed down in the *daf-2(e1370)* mutant, but blebbing was not; (2) inhibiting proliferation of germ cells prevented blebbing, but did not prevent convolution. Uncoupling of blebbing and other abnormal morphologies is not restricted to aged *C. elegans*. Previously, Chen et al. showed that fibroblasts lacking LamB1 or both LamB1 and LamB2 acquired more nuclear blebs but showed no changes in overall nuclear shape, whereas fibroblasts lacking all lamin genes (lamA/B1/B2) had more irregularly shaped nuclei but no change in nuclear blebbing frequency (Chen *et al.* 2018). Also, Coffinier et al. found that in cortical neurons of mice, LmnB1 deficiency induced nuclear blebbing, but LmnB2 deficiency led to elongation of nuclei instead of blebbing (Coffinier *et al.* 2011). Thus, different types of abnormalities in nuclear shape may reflect distinct biological processes.

## Conclusions

In summary, we find that in *C. elegans*, the frequency of nuclear blebbing in hypodermal cells is not correlated with the rate of aging but is affected by proliferative germ line stem cells. Our findings suggest that somatic nuclear blebbing is not a biomarker of organismal aging in *C. elegans*.

## Supplementary Material

jkad029_Supplementary_Data

## Data Availability

Strains and plasmids are available upon request. The authors affirm that all data necessary for confirming the conclusions of the article are present within the article, figures and tables. Supplemental material available at G3 online.
