# Supplementary material for: Somatic nuclear blebbing in Caenorhabditis elegans is not a feature of organismal aging but a potential indicator of germline proliferation in early adulthood
Source: G3 (Bethesda). 2023 Feb 3;13(4):jkad029. doi: 10.1093/g3journal/jkad029 (PMC10085788; doi:10.1093/g3journal/jkad029)
Supplement: jkad029_Supplementary_Data [file jkad029_supplementary_data.zip › File_S2_G3-2023-404061.docx]

Modeling:

In an 8-hour observation experiment of nuclear blebs, we found that 1 in 97 blebs detached from the parent nucleus. Assuming that the number of blebs attached to the nucleus conform to

$$\begin{aligned} \frac{N_{t}}{N_{0}}=e^{-\frac{t}{\tau}}\#\left( 1 \right) \end{aligned}$$

where $N_{0}$ is the number of blebs attached to the nucleus at the beginning (i.e., $t=0$) and $N_{t}$ is the number of attached blebs after 8 hours (i.e., $t=8$)，we can calculate the coefficients $\tau$ as:

$$\begin{aligned} \tau=-\frac{t}{ln\left( \frac{N_{t}}{N_{0}} \right)}=-\frac{8}{\ln\left( \frac{96}{97} \right)}\approx772 \left( h \right)\#\left( 2 \right) \end{aligned}$$

For each nuclear bleb, its detachment probability at time $t$ is

$$\begin{aligned} G_{t}=1-\frac{N_{t}}{N_{0}}=1-e^{-\frac{t}{\tau}}\#\left( 3 \right) \end{aligned}$$

Thus, the detachment rate of each bleb will be

$$\begin{aligned} g_{t}=\frac{dG_{t}}{dt}=\frac{1}{\tau}e^{-\frac{t}{\tau}}\#\left( 4 \right) \end{aligned}$$

and the rate of retained blebs at time $t$ will be:

$$\begin{aligned} \frac{dR_{t}}{dt}=f_{t}-R_{t}g_{t}=f_{t}-R_{t}\frac{1}{\tau}e^{-\frac{t}{\tau}}\#\left( 5 \right) \end{aligned}$$

where $R_{t}$ and $f_{t}$ are the average ratio of blebs retained by each nucleus at time $t$ and the generation rate of blebs, respectively.

Further, we assume that the increase of $R_{t}$ follows a negative exponential function, as

$$\begin{aligned} R_{t}=1-C_{0}e^{-\frac{t}{\lambda}}\#\left( 6 \right) \end{aligned}$$

Moreover, we detected the ratio of remaining blebs at different time points (Fig. 1B in manuscript):

| Day | 1 | 2 | 3 | 4 | 6 | 9 | 14 |
| --- | --- | --- | --- | --- | --- | --- | --- |
| Hours | 24 | 48 | 72 | 96 | 144 | 216 | 336 |
| blebs/nuclei | 0.0176 | 0.0617 | 0.1223 | 0.1310 | 0.2571 | 0.2731 | 0.3665 |

Using the above detected ratios and the linear regression of the logarithm of Eq. (6), i.e. $ln\left( 1-R_{t} \right)=lnC_{0}-\frac{t}{\lambda}$, we obtained $\lambda\approx717.63 \left( h \right), C_{0}\approx0.9829$, and derived the following result from Eq. (5):

$$\begin{aligned} f_{t}=\frac{dR_{t}}{dt}+R_{t}g_{t}=\frac{1}{\lambda}e^{-\frac{t}{\lambda}}+\left( 1-{C_{0}e}^{-\frac{t}{\lambda}} \right)\frac{1}{\tau}e^{-\frac{t}{\tau}}=\frac{1}{\lambda}e^{-\frac{t}{\lambda}}+\frac{1}{\tau}e^{-\frac{t}{\tau}}-\frac{C_{0}}{\tau}e^{-\left( \frac{1}{\tau}+\frac{1}{\lambda} \right)t}\#\left( 7 \right) \end{aligned}$$

Therefore, the ratio of blebs generated by each nucleus at time $t$ is:

$$\begin{aligned} F_{t}=\int_{0}^{t} f_{t}dt=2-\frac{C_{0}\lambda}{\lambda+\tau}-e^{-\frac{t}{\lambda}}-e^{-\frac{t}{\tau}}+\frac{C_{0}\lambda}{\lambda+\tau}e^{-\left( \frac{1}{\tau}+\frac{1}{\lambda} \right)t}\#\left( 8 \right) \end{aligned}$$

and the ratio of detached blebs for each nucleus at time $t$ is:

$$\begin{aligned} \tilde{G}_{t}=\int_{0}^{t} R_{t}g_{t}dt=1-\frac{C_{0}\lambda}{\lambda+\tau}-e^{-\frac{t}{\tau}}+\frac{C_{0}\lambda}{\lambda+\tau}e^{-\left( \frac{1}{\tau}+\frac{1}{\lambda} \right)t}\#\left( 9 \right) \end{aligned}$$

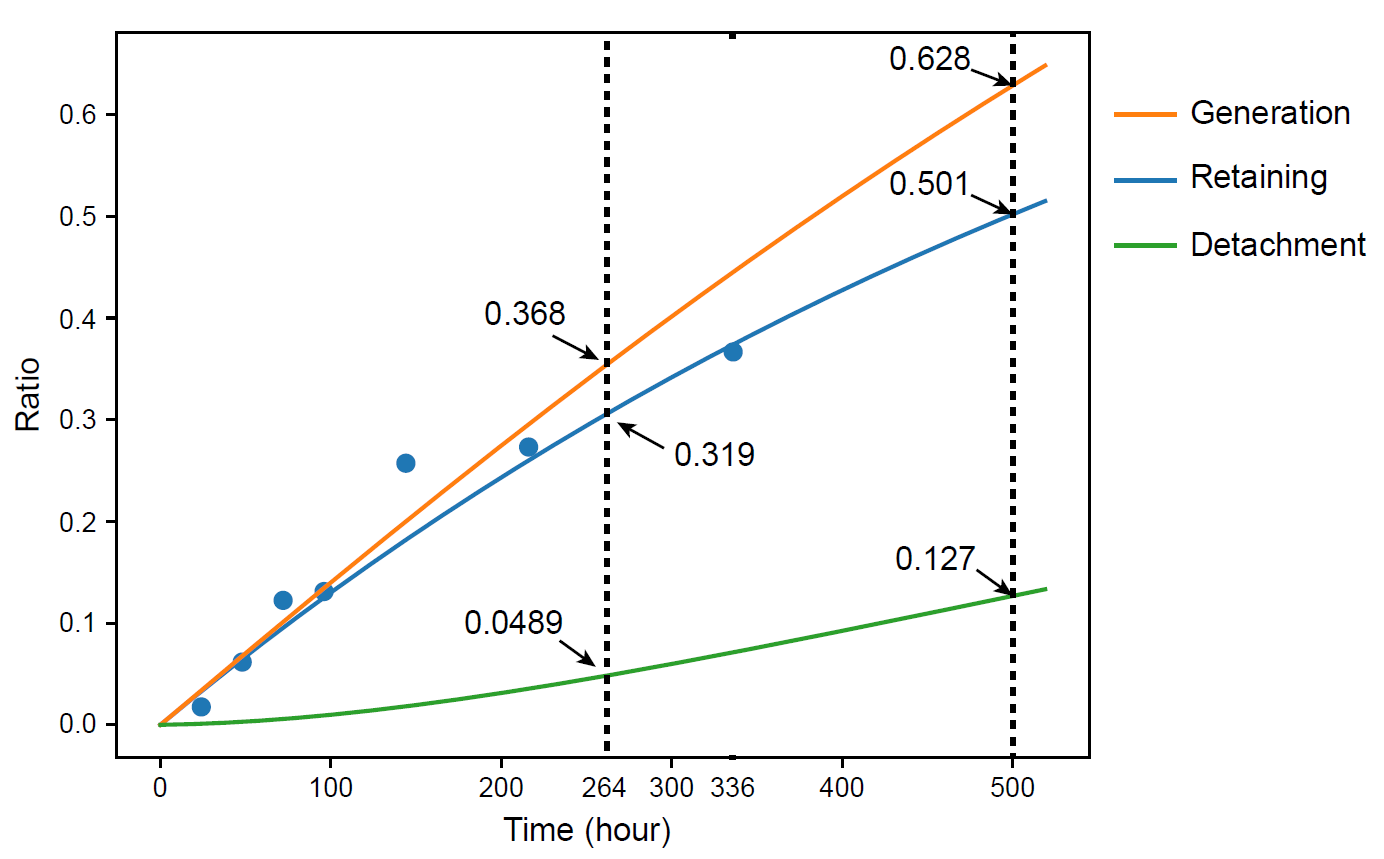


Figure S1 in File S2: Regression results for bleb generation ratio ($F_{t}$), detachment ratio ($\tilde{G}_{t}$), and retaining ratio ($R_{t}$). Blue dots: observation results; orange line: $F_{t}$; blue line: $R_{t}$; green line: $\tilde{G}_{t}$. Pearson correlation coefficient between observation results and $R_{t}$ is 0.970.
