# Supplementary material for: Somatic nuclear blebbing in Caenorhabditis elegans is not a feature of organismal aging but a potential indicator of germline proliferation in early adulthood
Source: G3 (Bethesda). 2023 Feb 3;13(4):jkad029. doi: 10.1093/g3journal/jkad029 (PMC10085788; doi:10.1093/g3journal/jkad029)
Supplement: jkad029_Supplementary_Data [file jkad029_supplementary_data.zip › Supplemental_Material_Legends_G3-2023-404061.docx]

**Supplemental Figure Legends**

Fig S1, related to Fig 3. Chromatin loss during aging

(A) qPCR results verifying that gene copy numbers decreased during aging as reported previously (Golden et al. 2007). The data shown are the mean ± standard error from two independent biological replicates. (B) The level of histone H3 decreased during aging. The same number of worms were used for each sample. (C) γ-irradiation (IR) did not induce nuclear blebbing. ns, not significant (p > 0.00l), as determined by Student’s T-test.

Fig S2. FUDR-treated worms have no proliferating germline stem cells

(A) Germline of an untreated control worm. (B) Germline of a FUDR-treated worm. The presence of meiotic cells, oocyte, sperm and embryo, and the absence of proliferating stem cells are shown. Scale bars, 10 µm.

Fig S3, related to Fig 5. Nuclear blebbing is inhibited upon FUDR treatment in *emr-1::gfp* knock in strain

Nuclear blebbing frequency on AD5 in the *emr-1::gfp* knock-in strain. The *p* value of a Student’s T-test is indicated on the graph.

Fig S4. ThT-treatment ameliorates nuclear blebbing

On AD5, nuclear blebbing frequency was much lower in ThT treated worms. The hyp 7 nuclei of the *gfp::lmn-1* knock-in strain UD484 were imaged and analyzed. The *p* value of a Student’s T-test is indicated on the graph.
