# Supplementary figures and images for: Somatic nuclear blebbing in Caenorhabditis elegans is not a feature of organismal aging but a potential indicator of germline proliferation in early adulthood
Source: G3 (Bethesda). 2023 Feb 3;13(4):jkad029. doi: 10.1093/g3journal/jkad029 (PMC10085788; doi:10.1093/g3journal/jkad029)

A

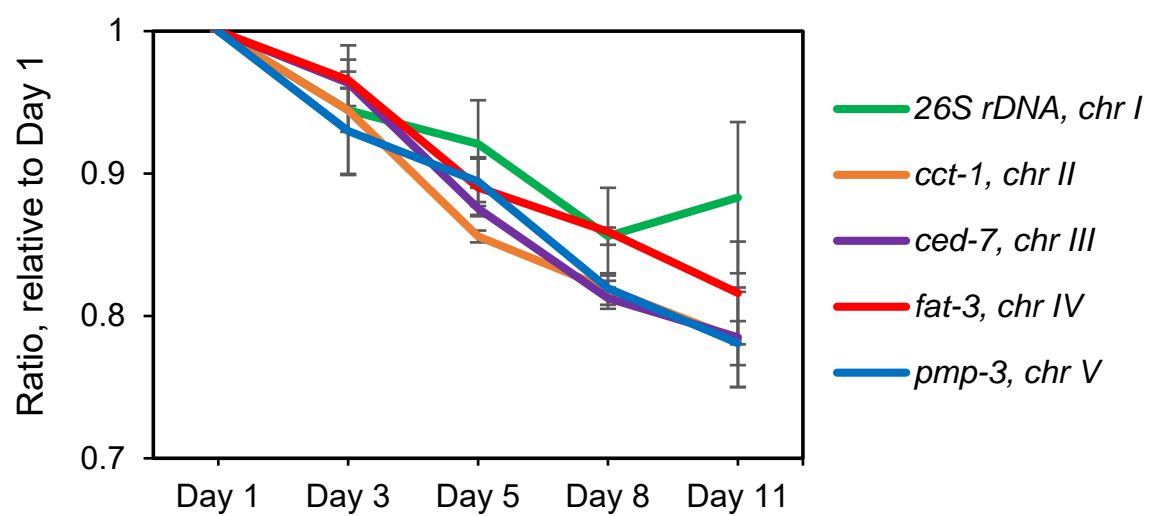

B

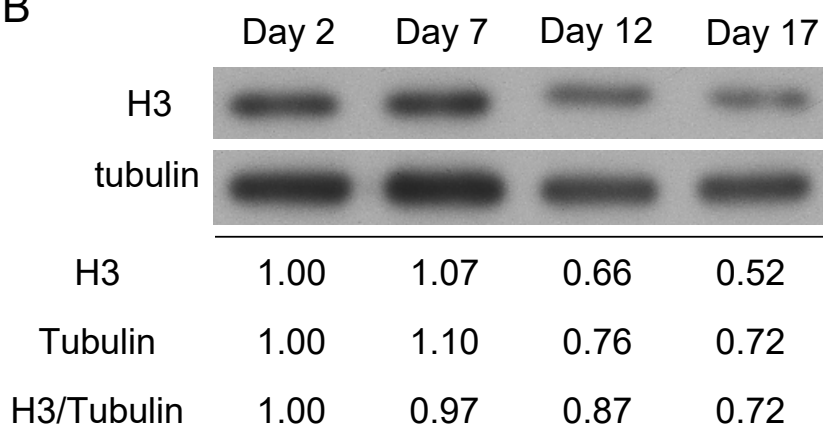

C

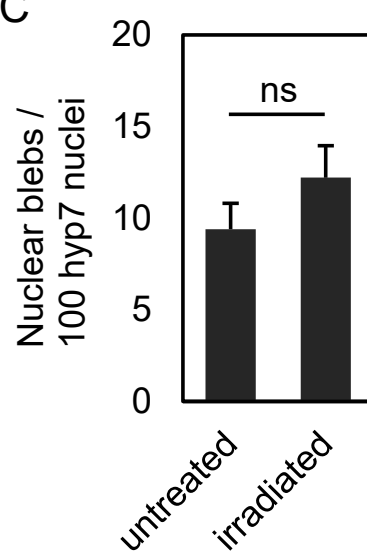

Supplement: jkad029_Supplementary_Data [file jkad029_supplementary_data.zip › Figure_S1_G3-2023-404061.pdf]

**A**

no FUDR

DAPI + EMR-1::GFP

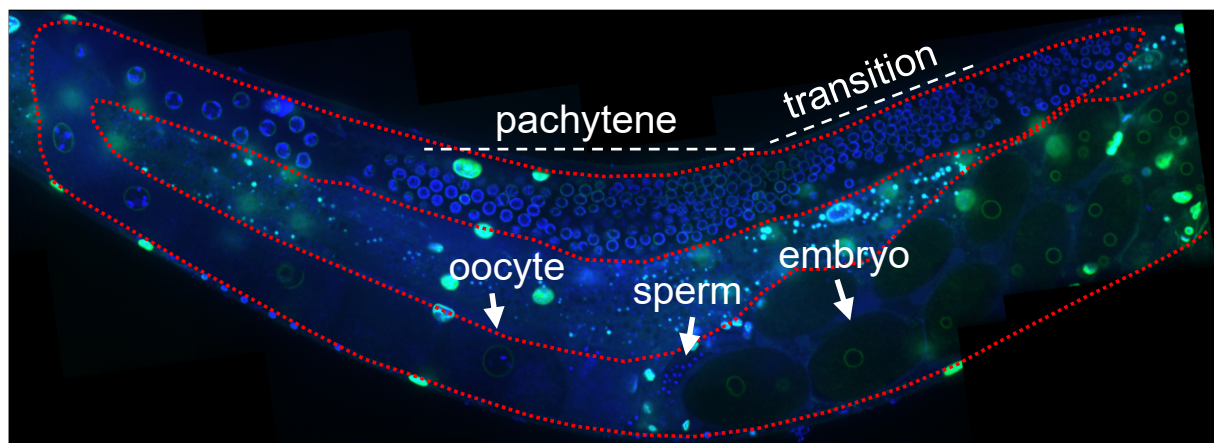

DAPI

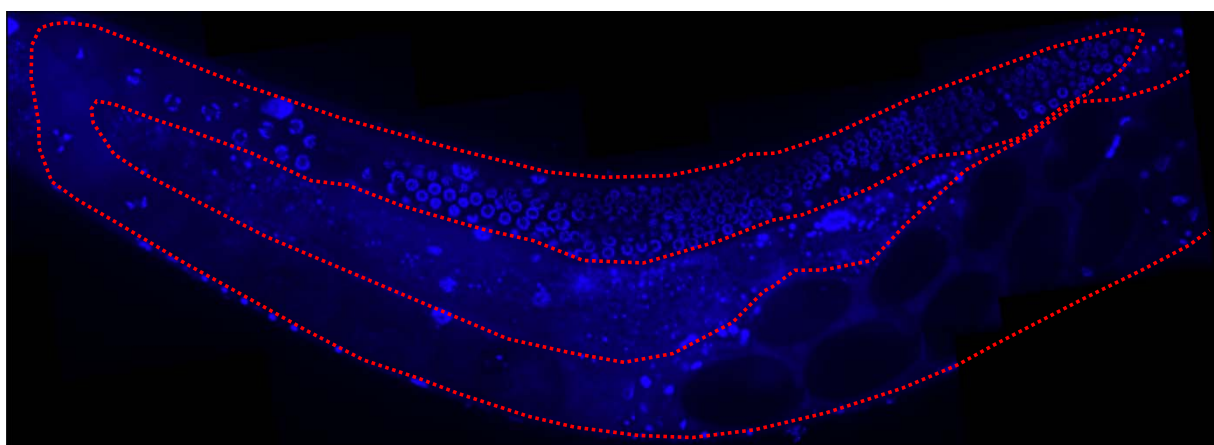

**B**

100  $\mu$ M FUDR

DAPI + EMR-1::GFP

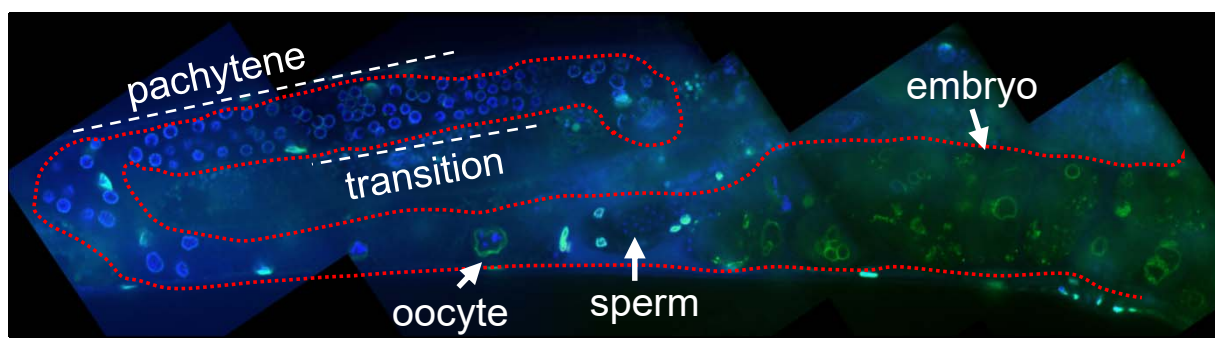

DAPI

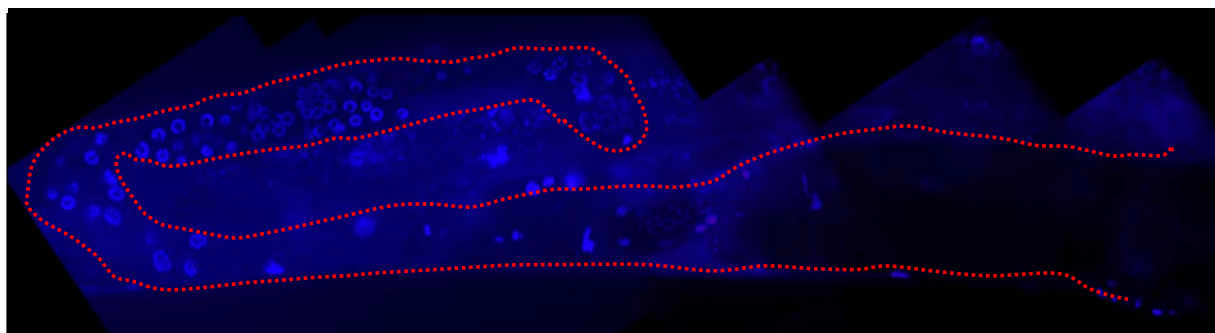

Supplement: jkad029_Supplementary_Data [file jkad029_supplementary_data.zip › Figure_S2_G3-2023-404061.pdf]

*emr-1::gfp*  
knock in strain

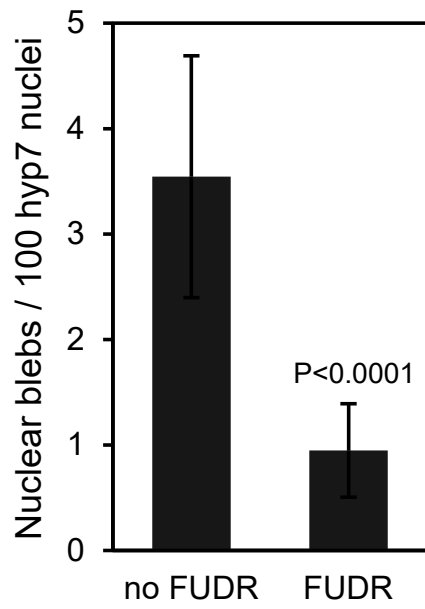

Supplement: jkad029_Supplementary_Data [file jkad029_supplementary_data.zip › Figure_S3_G3-2023-404061.pdf]

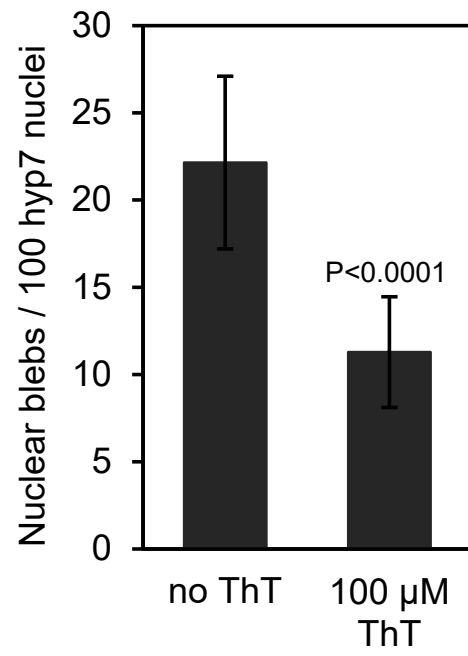

Supplement: jkad029_Supplementary_Data [file jkad029_supplementary_data.zip › Figure_S4_G3-2023-404061.pdf]

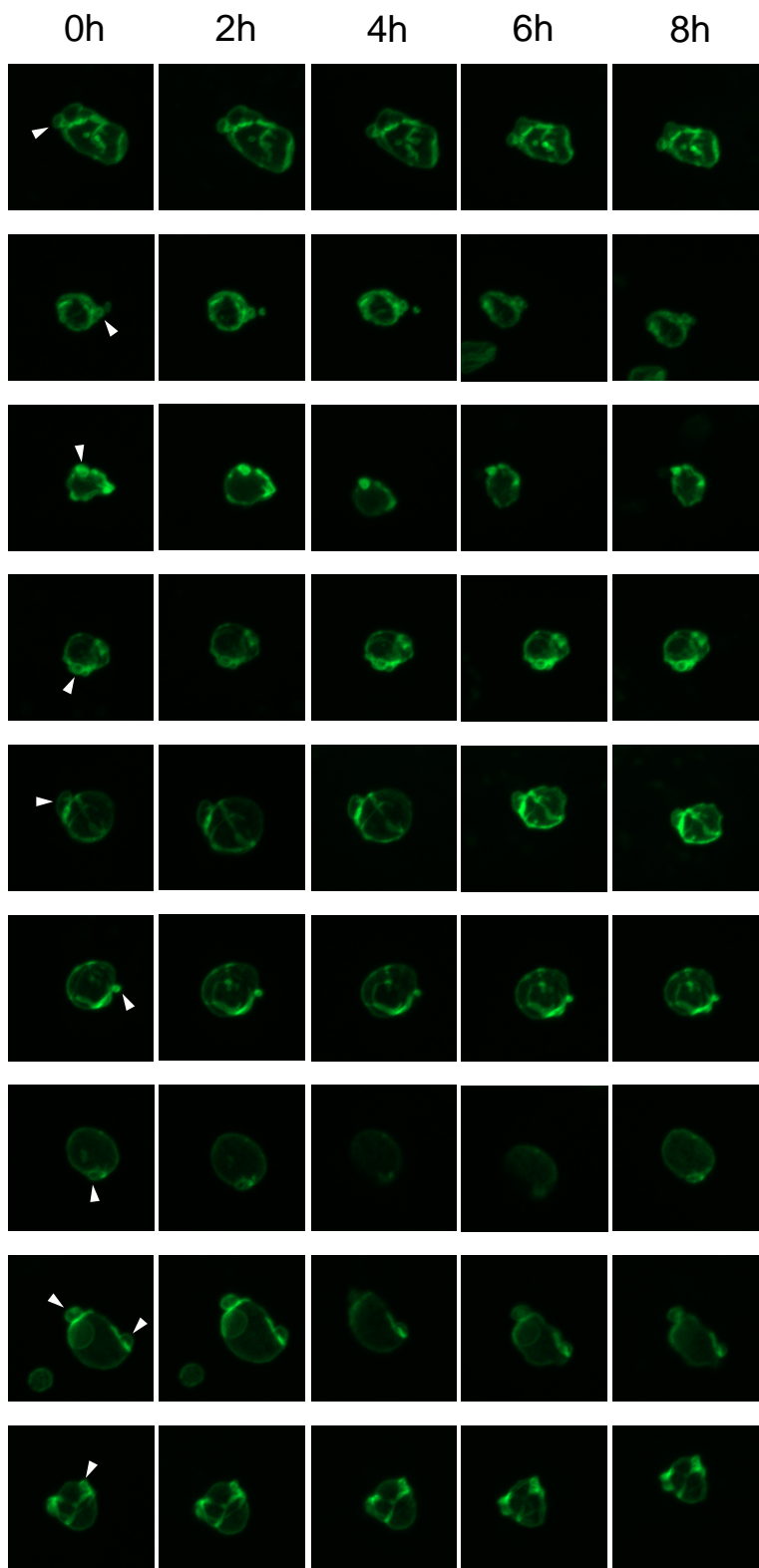

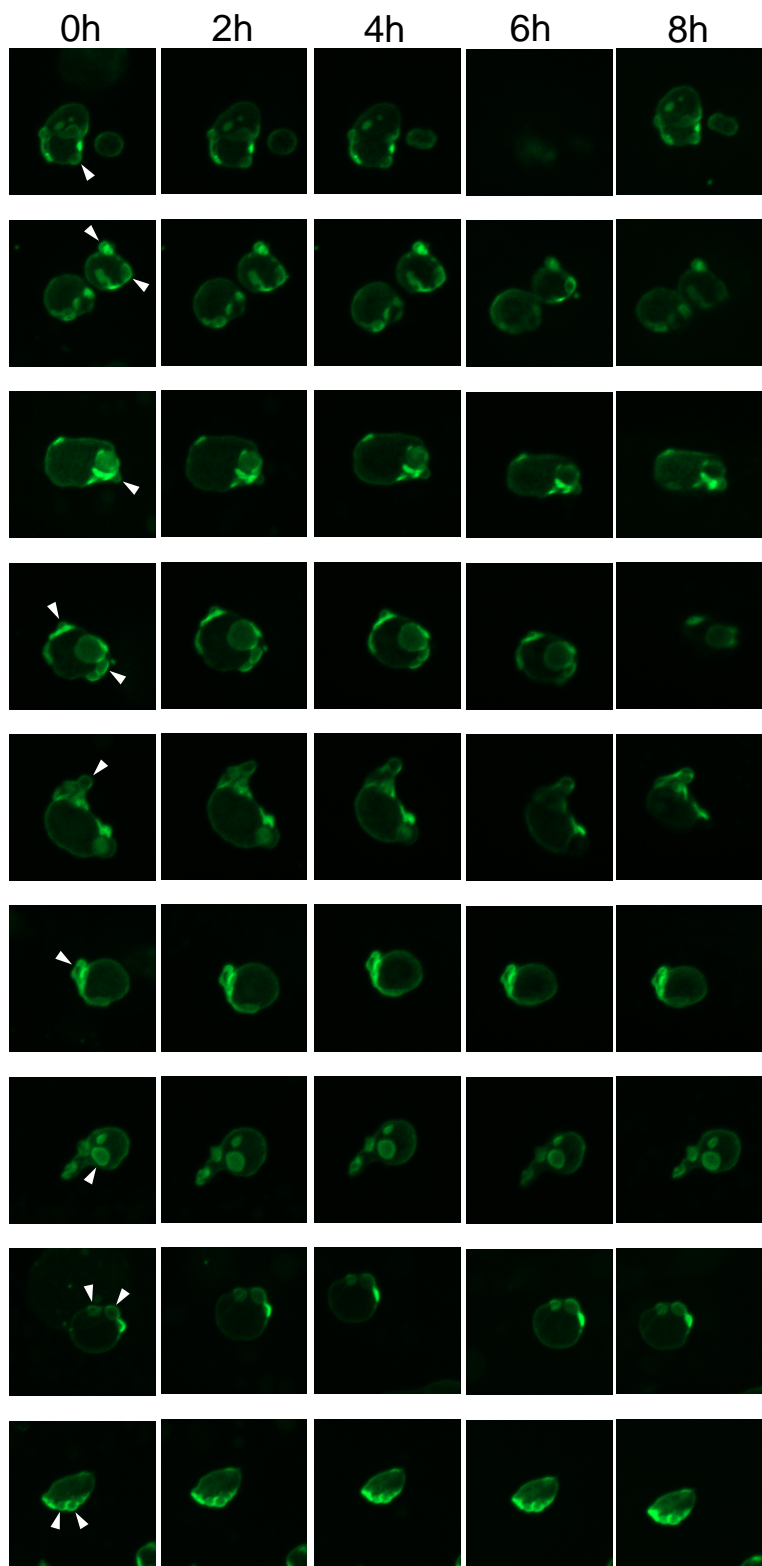

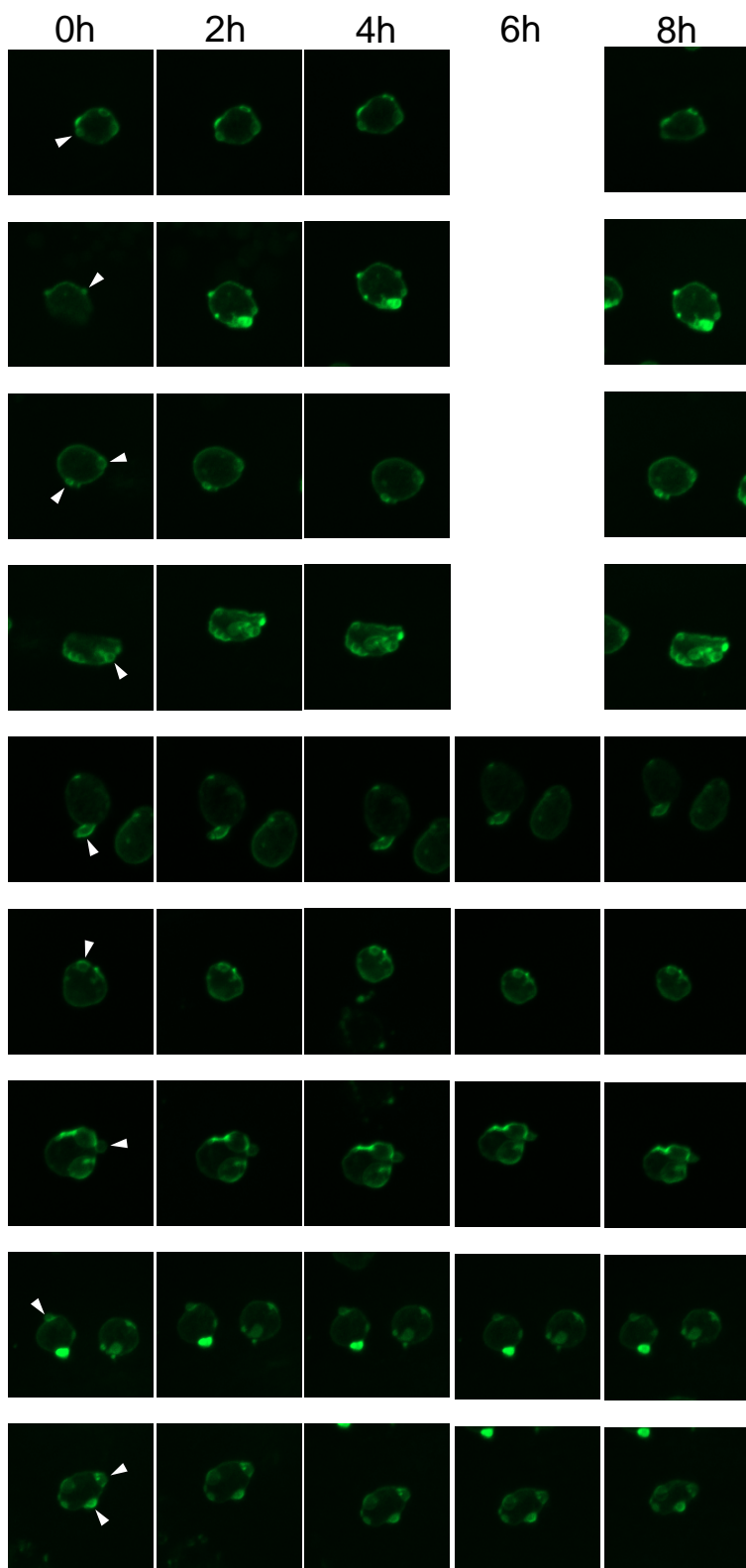

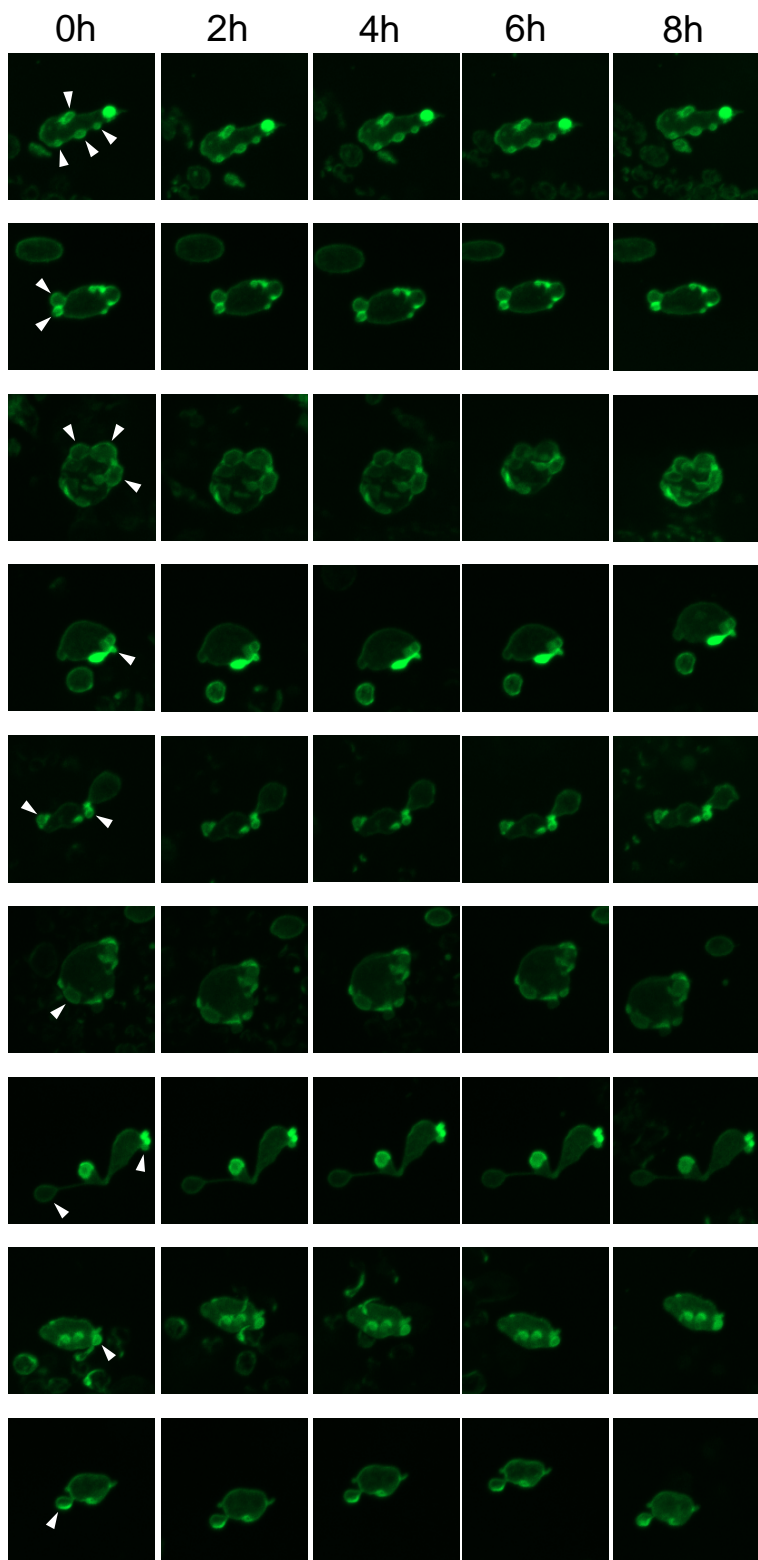

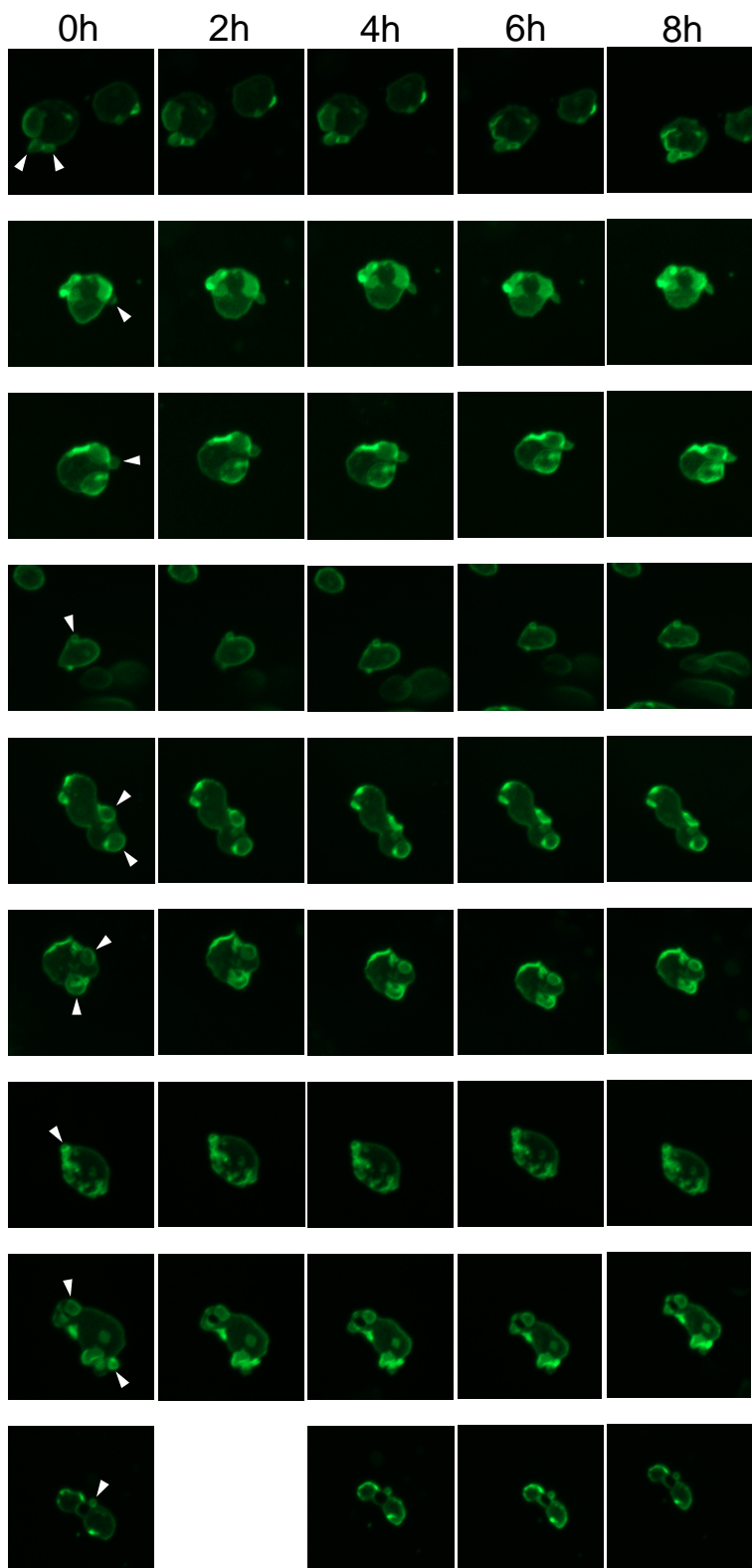

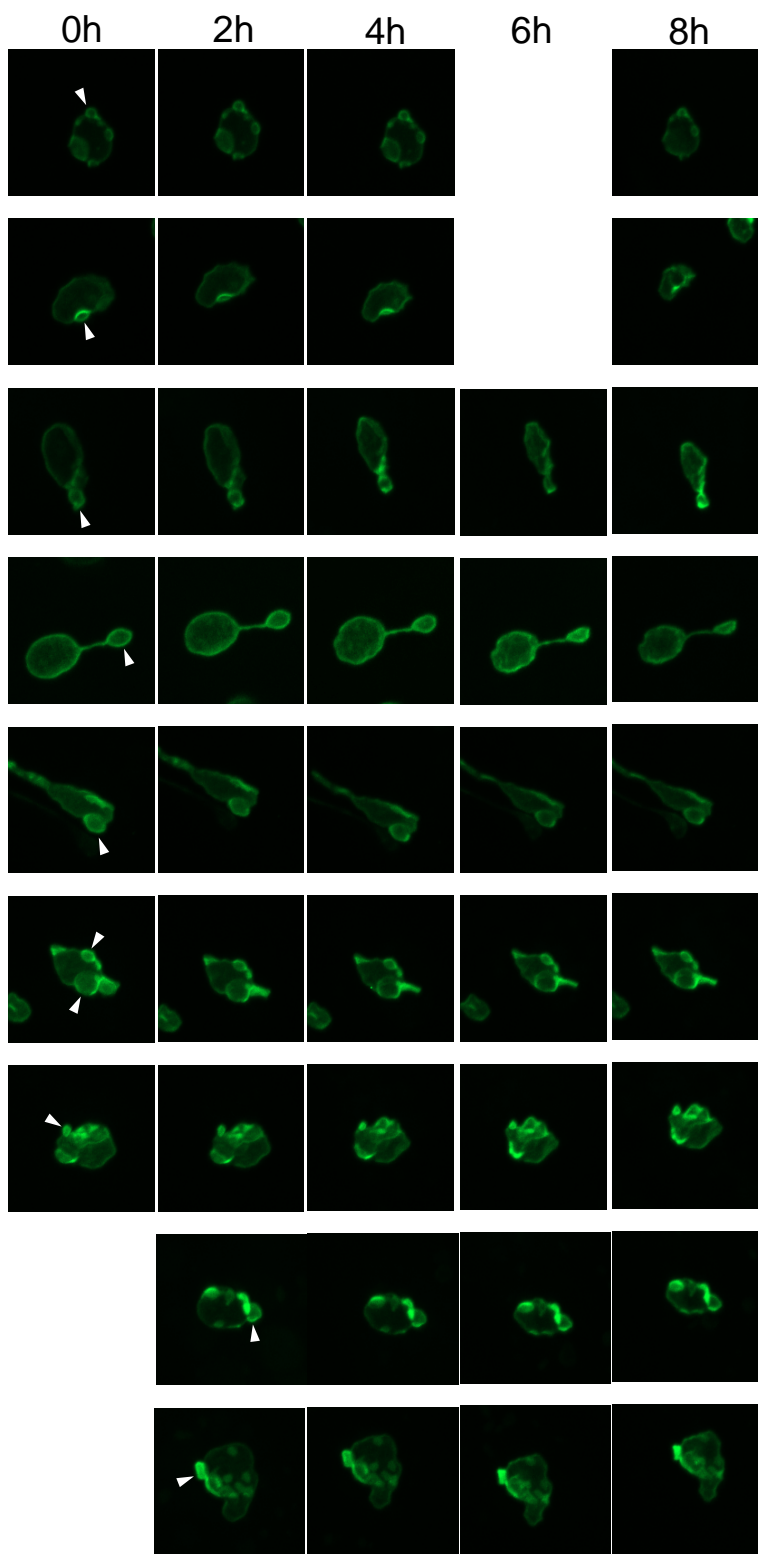

0h

2h

4h

6h

8h

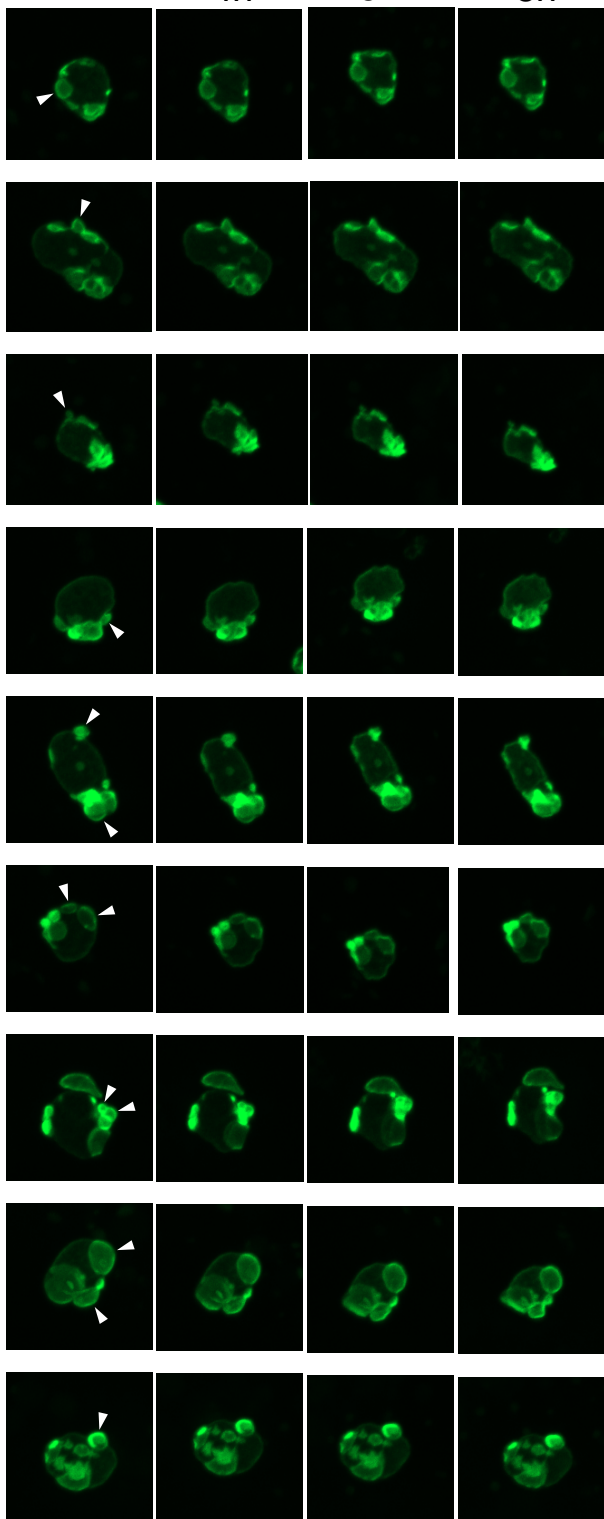

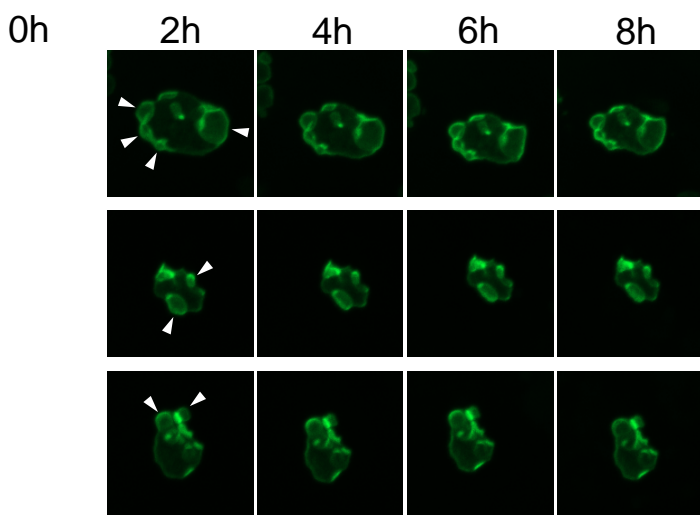

Figures in File S2: Timelapse imaging 95 nuclear blebs. Nuclear membrane are marked by EMR-1::GFP.

Supplement: jkad029_Supplementary_Data [file jkad029_supplementary_data.zip › File_S1_G3-2023-404061.pdf]
